# Improvements in pain, medication use and quality of life in onabotulinumtoxinA-resistant chronic migraine patients following erenumab treatment – real world outcomes

**DOI:** 10.1186/s10194-020-01214-2

**Published:** 2021-01-09

**Authors:** J. Talbot, R. Stuckey, L. Crawford, S. Weatherby, S. Mullin

**Affiliations:** 1 Southwest Neurology Audit and Research group (SoNAR), Department of Neurology, Derriford Hospital, Plymouth, PL6 8DH UK; 2 Peninsula Medical School, University of Plymouth, Plymouth, UK; 3 UCL Queen Square Institute of Neurology, University College London, London, UK

**Keywords:** Chronic migraine, Erenumab, Aimovig, OnabotulinumtoxinA, Botox, CGRP antagonist

## Abstract

**Background:**

The CGRP antagonists offer a novel therapeutic approach in migraine. Their utility in patients with severe forms of chronic migraine is a subject of particular interest. We present outcomes of 9 months of erenumab treatment in a cohort of patients with difficult-to-control chronic migraine, all of whom had prior unsatisfactory response to onabotulinumtoxinA.

**Methods:**

We offered erenumab to 98 patients with a prior unsatisfactory response to onabotulinumtoxinA. Eighty of 98 had trialled greater occipital nerve injections (82%), 32/98 peripheral neurostimulation (33%) and 18/98 intravenous dihydroergotamine (18%). Thirty eight of 98 (39%) met the definition of triptan overuse and 43/98 (44%) analgesic overuse. All patients met the EHF criteria for ‘resistant migraine’. Outcome measures (recorded monthly) included days with headache limiting activities of daily living (“red”), not limiting (“amber”), headache free (“green”), and requiring triptans or other analgesics. Quality of life scores - headache impact test 6 (HIT-6), patient health questionnaire 9 (PHQ-9) and pain disability index (PDI) - were also measured.

**Results:**

Mean number of red days improved by − 6.4 days (SE 0.67, 95%CI − 7.7 to − 5.1, *p*=0.001) at 3 months; − 6.8 days (SE 0.96, 95%CI − 8.80 to − 4.9, *p*=0.001) at 6 months and − 6.5 days (SE 0.86, 95%CI − 8.3 to − 4.8, *p*=0.001) at 9 months. Repeated measures ANOVA confirmed improvements in the number of red (*p*=0.001), green (*p*=0.001), triptan (*p*=0.001) and painkiller days (*p*=0.001) as well as scores of the HIT-6 (*p*=0.001), PHQ-9 (*p*=0.001), and PDI (*p*=0.001) across the duration of study.

**Conclusion:**

We observed improvements in pain, medication use and quality of life in onabotulinumtoxinA-resistant chronic migraine patients following erenumab treatment.

**Supplementary Information:**

The online version contains supplementary material available at 10.1186/s10194-020-01214-2.

## Background

The calcitonin gene-related peptide (CGRP) monoclonal antibodies offer a novel therapeutic approach to migraine. Their efficacy has been demonstrated in clinical trials of both acute and chronic migraine [1–10]. In 2017, a randomised controlled trial of erenumab in chronic migraine demonstrated a reduction of between − 1.4 and − 3.5 migraine days at 9 to 12 weeks compared to placebo [8]. In 2018, erenumab was approved by both the US Food and Drug Administration (FDA) and the European Medicines Agency (EMA) for episodic migraine and chronic migraine in adults who have at least 4 migraine days per month.

Emerging real-world data suggests that erenumab can be effective in patients with more severe migraine phenotypes, including those with a history of treatment failure [11–23]. Its utility in patients with a prior unsatisfactory response to onabotulinum toxinA (BoNTA), is of particular interest. In view of the relatively higher cost of this treatment compared to oral preventatives and its requirement to be administered in a clinic by trained healthcare providers, it is generally reserved for patients with more severe forms of migraine.

The emergence of CGRP antagonists as a novel migraine treatment has created considerable interest amongst clinicians, however associated costs of treatments currently limit their use in clinical practice. In England, for example, erenumab is deemed not to be cost effective within its marketing authorisation, and remains unavailable for patients. A recent appeal against this decision upheld that the National Institute of Clinical Excellence (NICE) had failed to consider the potential benefit of erenumab specifically in one group of patients; those that had failed to benefit from BoNTA or when it is contra-indicated (https://www.nice.org.uk/guidance/gid-ta10302/documents/appeal-decision).

In view of this, we present our outcomes of erenumab treatment (provided under a commercial supply agreement) in an open-label audit of patients with chronic migraine, all of whom had a prior unsatisfactory response to BoNTA.

## Methods

The study was designed and conducted by the neurology department at Derriford Hospital, Plymouth, a large tertiary referral centre for headache treatment in Devon and Cornwall, United Kingdom. It was registered locally as an audit, which under current national guidelines does not require research ethics committee review (http://www.hra-decisiontools.org.uk/research/).

### Participants and setting

Between February 2019 and July 2020 erenumab was offered to patients who met the International Classification of Headache Disorders (ICHD) definition of chronic migraine. All had previously had an unsatisfactory response to BoNTA (≤30% reduction in headache days following two treatments and/or lack of tolerability) and had failed and/or had contraindications to ≥2 classes of preventative medications. Patients with medication overuse were included. Patients receiving oral prophylactic medications were allowed to continue this during the study. No patient received BoNTA concurrently with erenumab.

Invitation letters were sent to patients deemed eligible: Responders were booked into an information session conducted by the headache specialist nurse (RS) supported by the headache assistant (LC). During this session patients received verbal and written information about the medication, training in self-administration of subcutaneous injections, further instruction in how to fill out the headache diaries and were individually consented and signed a written consent form. This specified the possibility that the drug might be stopped following discontinuation of the ‘free of charge scheme’ and that data would be collected in order to determine their treatment response.

Erenumab was provided free of charge by Novartis (The Westworks, 195 Wood Lane, London, W12 7FQ) through a commercial supply agreement. The drug was delivered directly to participants. Participants were free to discontinue at any point. In order to objectively document treatment response, data relating to the number of headaches pre and post treatment as well as effect on quality of life was collected. They received telephone follow-up via the unit headache nurse (RS) at 2 months to document side effects and assess treatment response and at 3 monthly intervals thereafter.

All patients started on a dose of 70 mg, self-injecting monthly. At the first telephone follow up, patients were offered a dose increase from 70 mg to 140 mg unless they were experiencing side effects deemed to be related to erenumab and which contraindicated such an increase. Those who did not receive a dose escalation at 2 months were reassessed at each subsequent follow up and were offered a dose increase if appropriate.

### Outcome measures

Patients were asked to record a standardised (handwritten) headache diary. A traffic light scoring system of the number of ‘red’, ‘amber’ and ‘green’ days was used to grade headache severity. Red days represented days with headaches which limited activities of daily living or which required use of triptans, amber days represented days with headaches but no limitation to activities of daily living, and green days represented headache-free days. Patients also completed standardised, validated scores of various functional domains – patient health questionnaire 9 (PHQ-9) [24], headache impact test 6 (HIT-6) [25, 26] and pain disability index (PDI) [27], and recorded the number of days requiring triptans and requiring other painkillers (simple and/or opiate-based). All data was returned monthly via post or email.

### Statistics

Graphs and statistical analyses were performed on Stata version 14.0 (4905 Lakeway Drive, Texas, 77,845, USA). We examined the different reasons for discontinuing treatment by performing a sensitivity analysis. It revealed an association between discontinuation of erenumab and a poor treatment response/side effects. This meant that data was deemed to be not missing at random, and therefore imputation or other adjustment strategies would not be appropriate.

The normality of the distribution of the change in number of red days was assessed by visual inspection (q plots available in supplementary materials). As such, parametric analyses were deemed appropriate. A two tailed paired t-test was performed to assess the change in the number of red headache days from baseline (month 2) reported at 3 months, 6 months and 9 months from the commencement of treatment. A repeated measures ANOVA was used to assess the change in all scores across the 12 months of data collection.

We modelled the potential impact of missing data. We assumed that in those where missing data was present, there was a 10 day increase in the number of red days from baseline. Accordingly all missing values were set at + 10 red days from the baseline value (month 2). We reassessed the normality of these distributions and found they were non-parametric; we therefore repeated our repeated measures analysis using Friedman’s test and our cross sectional analyses using a Wilcoxon signed rank test.

A Bonferroni correction for multiple comparisons (factor of 11) was performed on all analyses.

## Results

### Participants

**Fig. 1:**
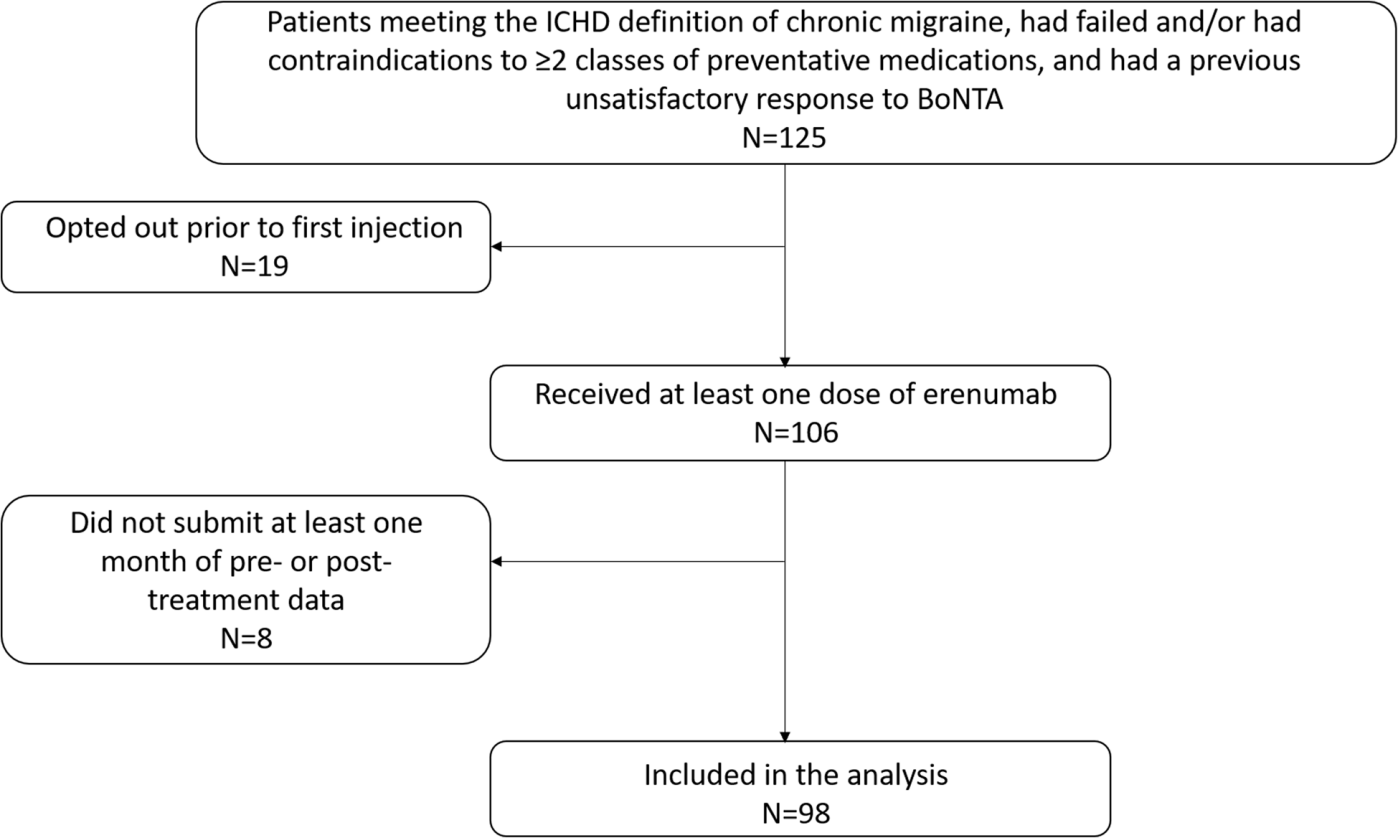
Audit design flowchart. 125 patients who met the ICHD definition of chronic migraine, had failed and/or had contraindications to ≥2 classes of preventative medications, and had a previous unsatisfactory response to BoNTA were selected for the audit. 19 patients did not attend the training day or opted out of the audit prior to the first injection. A total of 106 patients received at least one dose of erenumab. 8 patients who did not submit at least one month of pre- or post-treatment data were excluded from the analysis

A flow chart of recruitment is available in Fig. 1. One hundred and twenty five patients were sent invitation letters. Nineteen patients did not attend the training day or opted out prior to their first injection. A total of 106 patients received at least one dose of erenumab. Of these, 8 patients did not submit a single month of either pre- or post-treatment data and were not included in data analysis. Seven of 8 (88%) had no response to treatment, two of whom experienced side effects. In total data from 98 patients were analysed. Fifty six patients of these 98 (57%) underwent a dose increase to 140 mg at a median of 2 (IQR 1) months after treatment initiation.

A summary table of demographics at baseline are displayed in Table 1. Eighty one out of 98 (83%) participants were female. The mean age at enrolment was 50.4 (SD 12.4) years. Subjects had suffered with migraine for a mean of 19.6 years (SD 13.0). Of the 98 participants, 92/98 (94%) suffered ‘a severe impact’ from their headaches as determined by a HIT-6 score ≥60 [25], 81/98 (83%) had concurrent depression (PHQ-9 score ≥10) [24], 38/98 (39%) met the definition of triptan overuse (≥10 days/month) and 43/98 (44%) met the definition of analgesic overuse (≥15 days/month). All patients met the definition of ‘resistant migraine’ as defined by the recent European Headache Federation (EHF) consensus. As this was the first trial of a CGRP inhibitor in all our patients, none met the definition of ‘refractory migraine’, which stipulates a failed trial of this medication class [28].

**Table 1:** Demographic factors in patients receiving erenumab

Participant characteristics	
%female	81/98 (82.7%)
Mean age, SD (range)	50.4, 12.4 (18–75)
Mean, SD (range) number of years with migraine	19.6, 13 (3–58)
Mean, SD (range) number of trialled migraine-specific oral preventive drugs	5.5, 1.8 (1–9)
Previous trial of greater occipital nerve injections	80/98 (81.6%)
Previous trial of intravenous dihydroergotamine	18/98 (18.4%)
Previous trial of peripheral neurostimulation device	32/98 (32.7%)
Depression (based on PHQ-9 score >=10)	81/98 (82.7%)
Severe impact of headaches (HIT-6 >=60)	92/98 (93.8%)
Triptan overuse (>= 10 days/month)	38/98 (38.8%)
Other analgesic overuse (>=15 days/month)	43/98 (43.9%)
‘Resistant migraine’ by EHF criteria	98/98 (100%)

All patients had undergone treatment with BoNTA but had not achieved a clinically relevant response (≥30% improvement in headache days according to headache diaries/clinical assessment). Of the 98 patients, 80/98 (82%) had received a trial of greater occipital nerve injections, 18/98 (18%) had received a trial of intravenous dihydroergotamine and 32/98 (33%) had received a trial of a peripheral neurostimulation device.

We also quantified previous use of migraine-specific oral preventives, for which comprehensive information was available in 92/98 (94%) patients. The mean number of trialled migraine-specific oral preventives (including medications within the same class) in these patients was 5.5 (SD 1.8), which included beta-blockers (propranolol, atenolol; 68/92 patients, 74%), tricyclics (amitriptyline and nortriptyline; 80/92 patients, 87%), anticonvulsants (topiramate, gabapentin, pregabalin and sodium valproate; 83/92 patients, 90%), angiotensin II receptor blockers (candesartan; 47/92 patients, 51%), calcium channels blockers (flunarizine; 16/92 patients, 17%), serotonin antagonists (pizotifen; 39/92 patients, 42%), and anti-depressants (venlafaxine, mirtazapine, duloxetine; 8/92 patients, 9%).

### Safety, tolerability and missing data

A flow chart of study recruitment and retention is provided in Fig. 1. A table of missing data and reasons for this are included in Table 2. A total of 24/98 (24%) patients opted to discontinue erenumab during the study period: Eighteen of 98 (18%) patients chose to discontinue due to a perceived lack of benefit, and a further 5/98 (5%) discontinued due to one or more side effect(s), namely rash (1/98), palpitations (1/98), gastrointestinal upset (2/98), tightness in throat (1/98), and hypertension (1/98). One patient (1/98) became pregnant during treatment resulting in discontinuation of the drug. Forty-two of the 98 participants (43%) had not completed 9 months of treatment at the time of data analysis, but continued to take and tolerate erenumab.

**Table 2:** Table of missing data

Month of study	no treatment		baseline	treatment								
	0	1	2	3	4	5	6	7	8	9	10	11
**Total number of patients included in analysis**	71	93	98	97	90	86	83	72	50	45	31	27
**Data not submitted**	27	5	0	1	0	0	0	0	3	3	5	3
**Ineffective (cumulative)**	0	0	0	0	3	6	8	11	17	18	18	18
**Side effects (cumulative)**	0	0	0	0	3	4	4	5	5	5	5	5
**Other reason (cumulative)**	0	0	0	0	0	0	0	0	0	0	0	1
**Ongoing treatment at time of analysis (cumulative)**	0	0	0	0	2	2	3	10	23	27	39	44

Table accounting for missing data throughout the study period. Three months of baseline data (month 0, 1, 2) is followed by nine months of post-treatment data (month 3–11), when patients received monthly erenumab injections. The total number of patients included in the dataset for each month is shown at the top. This is broken down into the number of patients who failed to submit a questionnaire for each given month but remained in the analysis (‘data not submitted’), and cumulative discontinuation rates throughout the study period for various reasons (ineffectiveness, side effects, other (specifically, pregnancy). The final column shows the cumulative number of patients that were continuing to receive erenumab but had not completed sufficient months of treatment to provide data beyond a certain timepoint

### Outcomes

A table of outcomes across the period of data collection are provided in Table 3.

**Table 3:** Outcomes across the duration of the study

Month	Pre-treatment		Baseline	Post- treatment									ANOVA	
	0	1	2	3	4	5	6	7	8	9	10	11	Corr.	Uncorr.
**Red days**	15.2 (0.9, 7.7)	15.4 (0.8, 7.7)	15.7 (0.9, 8.2)	12.3 (0.9, 8.6)	10.6 (0.9, 8.4)	8.6 (0.73, 6.8)	8.6 (0.9,7.9)	7.8 (0.9,7.6)	6.7 (0.84,5.9)	5.7 (0.8,5.3)	5.0 (0.7,4.0)	4.9 (0.8,4.3)	*p*=0.001	*p*< 0.001
**Amber days**	10.9 (0.9, 7.9)	11.2 (0.8, 7.6)	11.0 (0.8, 7.6)	11.3 (0.9, 8.2)	11.1 (0.9, 7.9)	11.9 (0.7, 8.1)	11.3 (0.9, 8.5)	12.3 (1.0, 8.9)	11.6 (1.2, 8.7)	11.9 (0.7, 8.7)	11.7 (0.7, 8.9)	12.3 (1.8, 9.2)	*p*=1.000	*p*=0.985
**Green days**	3.3 (0.6, 4.7)	3.2 (0.5, 4.9)	3.6 (0.5, 5.4)	6.7 (0.8, 8.2)	8.4 (1.0, 9.2)	9.6 (1.0, 9.2)	10.4 (1.1, 9.6)	10.2 (1.1, 9.7)	11.6 (1.3, 9.0)	12.4 (1.4, 9.1)	13.4 (1.7, 9.7)	12.4 (1.9, 10.0)	*p*=0.001	*p*< 0.001
**Triptan days**	7.2 (0.9, 7.9)	7.7 (0.8, 8.0)	8.1 (0.9, 8.8)	5.7 (0.7, 7.2)	5.0 (0.7, 6.9)	4.6 (0.7, 6.0)	5.0 (0.8, 7.0)	4.5 (0.8, 6.4)	4.1 (0.7, 5.1)	4.1 (0.7, 4.8)	4.2 (0.8, 4.4)	4.2 (0.9, 4.9)	*p*=0.001	*p*< 0.001
**Painkiller days**	18.7 (1.2, 11.6)	14.7 (1.2, 11.4)	13.9 (1.2, 11.5)	13.0 (1.2, 11.0)	11.6 (1.1, 10.8)	11.3 (1.1, 10.2)	10.8 (1.1, 10.2)	10.2 (1.1, 10.1)	9.4 (1.4, 10.0)	8.5 (1.5, 10.0)	8.6 (1.8, 9.8)	9.1 (1.9, 9.8)	*p*=0.001	*p*< 0.001
**HIT6 score**	67.5 (0.7, 5.4)	66.6 (0.8, 8.0)	66.9 (1.0, 9.4)	62.4 (1.0, 10.4)	60.3 (1.2, 11.2)	59.1 (1.3, 11.7)	58.8 (1.2, 11.3)	60.0 (1.0, 8.5)	59.0 (1.1, 7.9)	58.6 (1.4, 9.2)	58.1 (1.9, 10.5)	57.5 (1.9, 9.7)	*p*=0.001	*p*< 0.001
**PHQ9 score**	16.7 (1.0, 7.8)	16.1 (0.7, 6.5)	16.5 (0.7, 6.4)	13.4 (0.7, 6.7)	12.1 (0.7, 6.7)	11.0 (0.7, 6.0)	10.5 (0.6, 5.8)	10.0 (0.7, 6.2)	9.6 (0.9, 6.1)	9.3 (0.9, 6.4)	9.2 (1.3, 7.0)	8.5 (1.2, 6.1)	*p*=0.001	*p*< 0.001
**PDI score**	44.1 (2.0, 16.6)	44.8 (1.7, 16.6)	45.8 (1.7, 16.8)	38.7 (2.0, 20.0)	35.1 (2.0, 19.1)	32.6 (2.3, 20.7)	33.3 (2.3, 20.7)	31.4 (2.4, 20.4)	29.4 (2.6, 18.3)	29.4 (3.2, 21.7)	28.0 (3.8, 20.8)	28.0 (4.3, 22.0)	*p*=0.001	*p*< 0.001
**t- test** **(Red days)**	**t value**					9.6			7.0			7.6		
	**Corrected**					*p*=0.001			*p*=0.001			*p*=0.001		
	**Uncorrected**					*p*< 0.001			*p*< 0.001			*p*< 0.001		

Values are mean (SE, SD)

Raw data for all outcomes. 98 patients received at least one dose of erenumab and were analysed. Three months of pre-treatment data were collected, with month 2 used as baseline. Outcomes are recorded up to nine months of treatment (month 11), and relate to the mean number of red days (headache limiting activities of daily living), amber days (headache but no limitation to activities of daily living), green days (headache-free), triptan days (requiring use of one or more triptans), painkiller days (requiring use of simple and/or opiate analgesics), HIT-6 (headache impact test-6) score, PHQ-9 (patient health questionnaire-9) score, and PDI (pain disability index) score. Analysis of variance *p* values are shown for each outcome, both corrected following Bonferroni calculation for multiple comparisons, and uncorrected. Below, t tests comparing mean scores relating to the change in number of red days per patient at month 5, 8 and 11 (three, six and nine months post-treatment, respectively) compared with baseline (month 2) are shown

### Effect on headache days

**Fig. 2:**
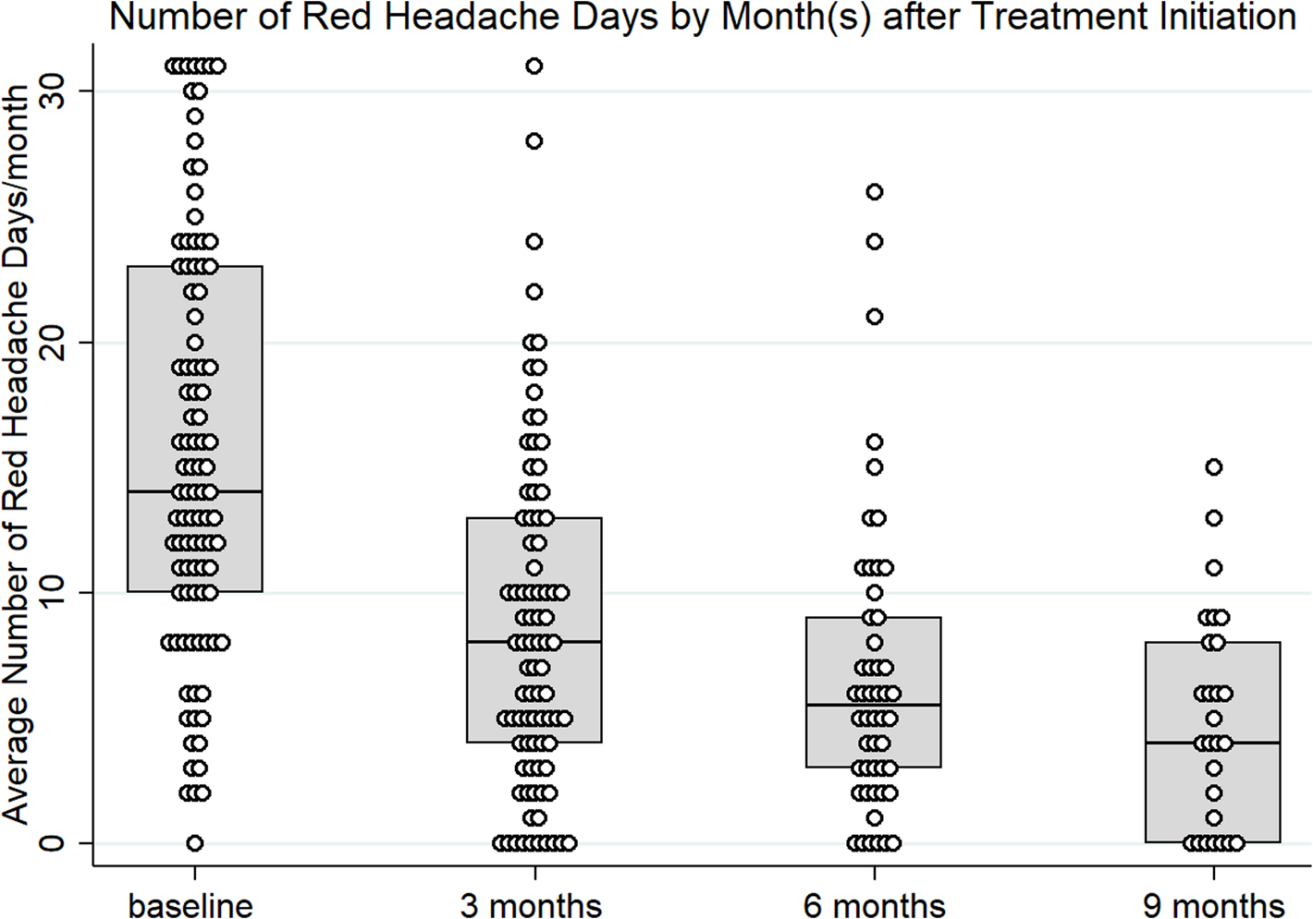
Box plots with dot plots, showing the number of red headache days/month at baseline (month 2) and 3, 6 and 9 months after treatment initiation (respectively months 5, 8 and 11)

**Fig. 3:**
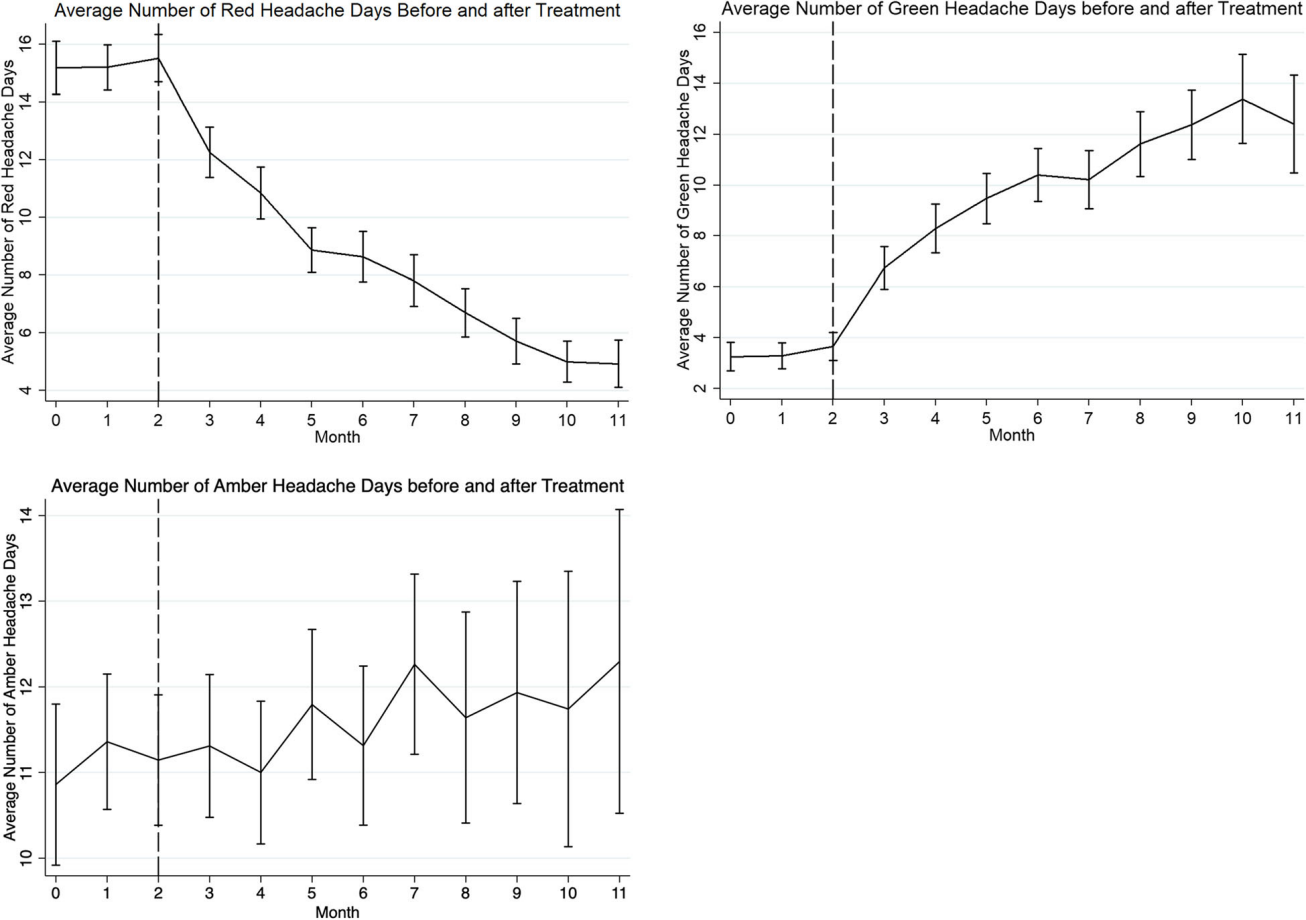
Graphs showing change in mean monthly headache days before and after commencing erenumab. Three months of baseline data (month 0, 1, 2) is followed by 9 months of post-treatment data (month 3–11). Red days signify days with headaches that limit activities of daily living, amber days signify days with headache but not limiting activities of daily living, and green days signify days free of any headache. Error bars represent standard error of the mean

There were sustained reductions in the mean number of red days per month across the study period (repeated measures ANOVA, *p*=0.001, Fig. 3). There was a mean of 15.7 (SD 8.2) red days/month at baseline. Following treatment there were improvements in monthly red days of − 6.4 days (SE 0.67, 95%CI − 7.7 to − 5.1, t=9.6, *p*=0.001, Fig. 2) at 3 months; − 6.8 days (SE 0.96, 95%CI − 8.8 to − 4.9, t=7.0, *p*=0.001), Fig. 2) at 6 months and − 6.5 days (SE 0.86, 95%CI − 8.3 to − 4.8, t=7.6, *p*=0.001, Fig. 2) at 9 months. Respectively 71/98 (72%), 42/50 (84%) and 25/27 (93%) experienced a reduction in the number of red days at 3, 6 and 9 months respectively. Fifty three of 90 (59%), 31/50 (62%) and 16/27 (59%) experienced at least a 5 day improvement (reduction) in the number of red days at 3, 6 and 9 months.

There was a mean of 3.6 (SD 5.4) green days/month at baseline. Following treatment there were improvements in the mean number of green days/month (repeated measures ANOVA p=0.001, Fig. 3). There were + 5.7 days (SE 0.8, SD 7.6) at 3 months, + 6.9 days (SE 1.1, SD 7.9) at 6 months and + 7.4 days (SE 1.5, SD 8.0) at 9 months. Forty six of 86 (53%), 28/50 (56%) and 17/27 (63%) respectively experienced some improvement in the number of green days at 3, 6 and 9 months. 36/86 (42%), 28/50 (56%) 15/27 (56%) experienced at least a 5 day improvement in the number green days at 3, 6 and and 9 months respectively. There was no significant difference in the number of amber days (repeated measures ANOVA *p*=1.0, Fig. 3).

### Effect on triptan and other painkiller use

**Fig. 4:**
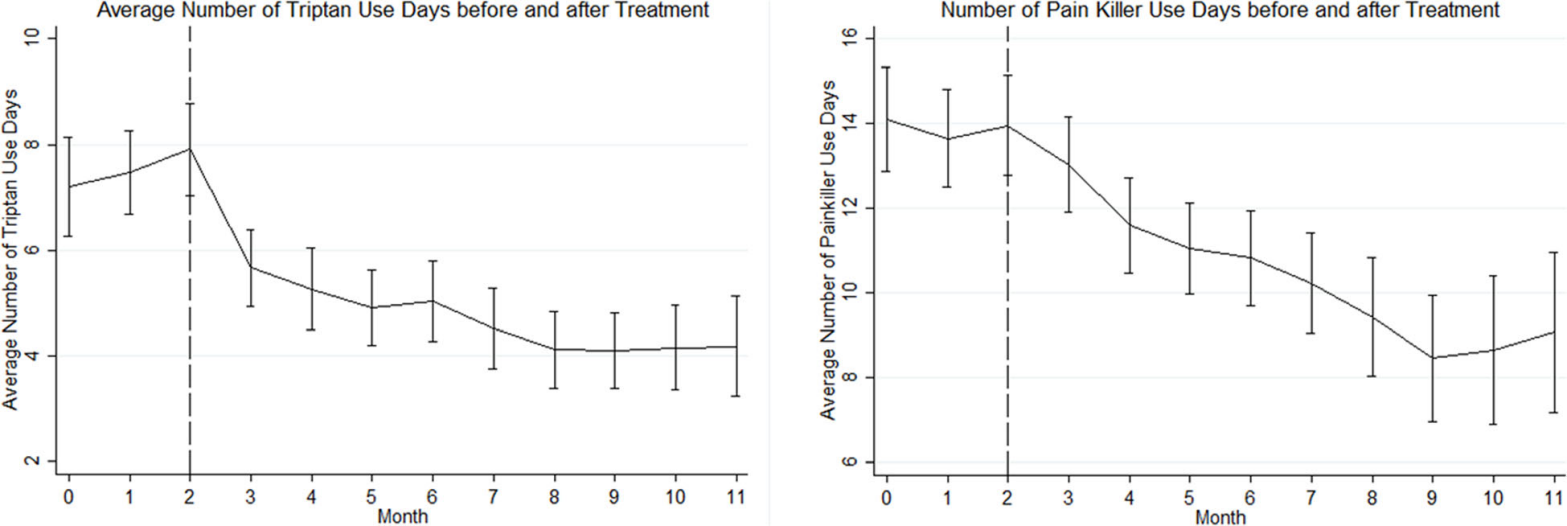
Graphs showing mean number of days requiring triptans, and requiring painkillers (simple or opiate analgesics), before and after commencing erenumab. Three months of baseline data (month 0, 1, 2) is followed by 9 months of post-treatment data (month 3–11). Error bars represent standard error of the mean

There were significant improvements in the mean number of days requiring triptans (repeated measures ANOVA, *p*=0.001, Fig. 4) and other painkillers (repeated measures ANOVA, *p*=0.001, Fig. 4). The change from baseline of mean number of days requiring triptans per month was − 3.4 days (SE 0.6, SD 6.0) at 3 months, − 3.9 days at 6 months (SE 0.9, SD 6.3) and − 3.9 (SE 1.0, SD 5.2) at 9 months. The mean number of painkiller days per month changed from baseline by − 2.2 days (SD 6.7) at 3 months, − 3.2 days (SD 7.9) at 6 months and − 3.9 days (SD 7.6) at 9 months. Of the patients who completed six months of treatment/assessments, 23/50 (46%) met the definition of triptan overuse (≥10 days/month) and 20/50 (40%) of analgesia overuse (≥15/month) at baseline. At 6 months 5/50 (10%) and 11/50 (22%) met the definition of triptan and analgesia overuse respectively.

### Effect on quality of life measures

**Fig. 5:**
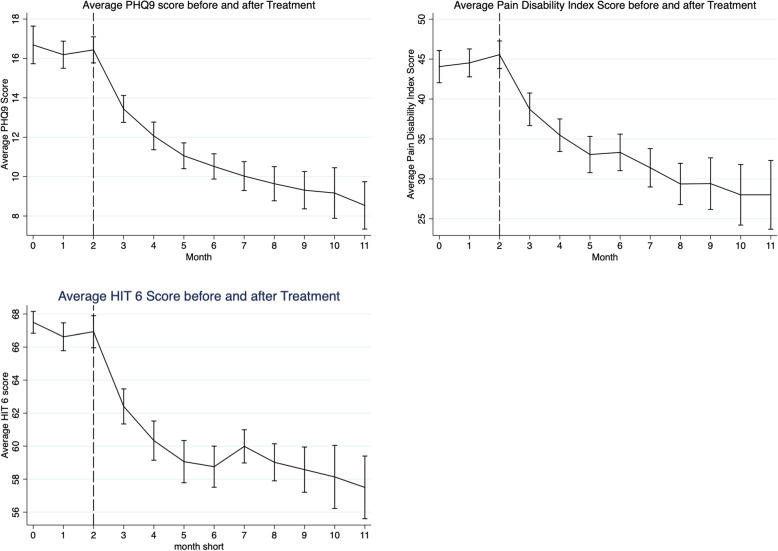
Graphs showing change in mean quality of life scores (PHQ9, HIT-6, and PDI) before and after commencing erenumab. Three months of baseline data (month 0, 1, 2) is followed by 9 months of post-treatment data (month 3–11). Error bars represent standard error of the mean

There were clinically meaningful [29–31] statistically significant improvements in the PHQ-9 (repeated measures ANOVA: *p*=0.001, Fig. 5), HIT-6 (repeated measures ANOVA: *p*=0.001, Fig. 5) and PDI (repeated measures ANOVA: *p*=0.001, Fig. 5). From baseline mean change in PHQ-9 score was − 5.2 (SE 0.62, SD 5.6) at 3 months, − 6.0 (SE 1.0, SD 7.1) at 6 months and − 7.2 (SE 1.1, SD 5.8) at 9 months. Mean change in HIT-6 score was − 7.1 (SE 1.1, SD 10.5) at 3 months, − 9.0 (SE 1.2, SD 8.7) at 6 months and − 10.9 (SE 1.8, SD 9.0) at 9 months. Mean change in PDI score was − 12.1 (SE 1.8, SD 16.0) at 3 months, − 14.6 (SE 1.0, SD 14.6) at 6 months and − 17.9 (SE 3.5, SD 18.0) at 9 months.

### Modelling to assess the impact of dropouts on change in number of red days

Because we were unable compensate for missing data statistically, we assessed the potential effect of dropouts using a crude and highly conservative model estimating the change of red headache days they were experiencing. Where data was missing, we stipulated that the number of red headache days increased by 10 days from the baseline (month 2) value. This meant that our model assumed each participant experienced a 10 day increase in the number of red days they were experiencing if missing data was present. Using this model, there was a significant change in the number of red days across the 12 months of data collection (Friedman’s test, *p*=0.001). A significant reduction in the number of red days remained at 3 months (median change − 5 days, IQR 10, Wilcoxon rank sign, *p*=0.001).

## Discussion

We present real world findings of erenumab treatment in ‘hard to treat’ chronic migraine sufferers. Our results show significant improvements in the number headache days, medication use and measures of functional performance. We feel the patient group is broadly representative of patients with difficult to control chronic migraine which frequently includes co-existing depression and medication overuse. All patients had an unsatisfactory response to BoNTA and had trialled multiple oral preventatives. A high proportion of patients had also received other treatment modalities.

The European Headache Federation (EHF) definitions of resistant and refractory migraine were recently revised [28]. Resistant migraine has been redefined as failure or contraindication to at least 3 specified classes of migraine preventatives and suffering from at least 8 debilitating headache days per month for at least 3 consecutive months without improvement. Refractory migraine relates to failure of all of the available preventatives, including BoNTA and a CGRP-modulating drug, and suffering from at least 8 debilitating headache days per month for at least 6 consecutive months. All patients in our cohort met the definition of resistant migraine at study enrolment.

A substantial majority (84% at 6 months) of our patient cohort reported a response to erenumab, which was in most cases relatively rapid. At 3 months post treatment there was a mean − 6.4 day improvement in the number of red days (headaches that impacted significantly on day-to-day activities) and a + 5.7 day improvement in the number of headache-free (green) days. At 6 months there was a mean − 6.8 day improvement in monthly red days and a mean improvement (+ 6.9 days) in the number of green days. Analgesia and triptan overuse both declined and there were clinically relevant improvements on all three measures of quality of life undertaken. We recorded outcomes up to 9 months in 27 patients. We found, in accordance with other studies [11, 12, 14, 20, 23], that patients continued to show sustained improvements over time, although the maximal benefit was seen in the first 4 months. This suggests that sustained trial of erunumab is necessary before being able to make a true assessment of treatment response.

A number of other studies have examined the effectiveness of erenumab in a non-controlled manner. An Italian study of erenumab in patients with enduring episodic and chronic migraine with high rates of medication overuse demonstrated a reduction of 15 migraine days in 76 patients [11]. Among the 44 patients who had failed treatment with BoNTA, 31 (70.5%) responded to the treatment. Giorgio et al. examined erenumab in treatment-refractory UK migraine sufferers that had failed ≥3 preventive treatments [12]. The majority had had an inadequate response to BoNTA and none had responded to greater occipital nerve blocks. They demonstrated significant reductions in monthly migraine days of 7.5 days at 6 months. A German study of erenumab in patients that had failed five oral prophylactics and type A botulinum toxin led to a reduction of 4.7 headache days after three treatment cycles [13]. A number of other recently published studies have shown similar improvements with erenumab [14–23], although only one has examined its benefit exclusively in BoNTA failures [18]. Our outcomes, recorded up to 9 months, in general exceed those of other studies [14–18, 23] and do not rely on retrospective reporting [13, 15, 21, 22], providing more objective evidence of treatment efficacy.

Our data suggests that erenumab is a safe and well-tolerated treatment. Only 5/98 (5%) discontinued erenumab due to side effects. This compares to 2.2% [11], 12% [12] and 4.3% [13] respectively in other comparable datasets. A large retrospective study of 241 patients treated with erenumab found an incidence of 70% of at least one adverse event, the most common being constipation, although most patients felt the benefit of treatment outweighed any drawbacks [21]. One patient in our cohort developed hypertension as a result of erenumab treatment, a complication noted previously [12], suggesting there is a need for monitoring of blood pressure in patients treated with erenumab.

### Limitations

We observed improvements in multiple outcomes following erenumab treatment, however as a non placebo-controlled study, it is not possible to infer causality. We also acknowledge the potential confounding effect of drop-outs and incomplete data. Our data probably overestimates the effect size of erenumab use on account of attrition bias, caused by preferential drop out of those who did not respond to the drug or experienced side effects. This is a limitation which is also a confounder in other comparable studies [11–13]. That said, our modelling demonstrates that even in the unlikely scenario that all those who dropped out (or had been treated for less than 9 months) experienced a 10 day increase in the number of monthly red days from baseline, significant reductions in the number of red days were retained. This suggests the confounding effect of attrition bias is unlikely to have resulted in type 1 error (false positive).

Our categorisation of headache differs to other studies, in which ‘migraine’ days are specifically defined, for example, on the basis of headache duration or a combination of pain and/or non-pain features. Our traffic light system was deliberately chosen to enable patients to categorise the severity and impact of their headache relatively easily, as we hoped this would potentially reduce subjective variation in reporting.

Post-hoc analysis of data from randomised studies of erenumab has suggested an advantage of the 140 mg dose on treatment outcomes, which is more evident in patients with a higher rate of prior treatment failure [32]. In our study the criteria for dose escalation was driven by patient preference, whilst dose increases were not uniformly undertaken at defined time points. As a result, the effect of 70 mg vs 140 mg dose in our cohort cannot be inferred, as this aspect of the dataset is subject to confounding and bias.

## Conclusions

We present our real-world findings of erenumab in a cohort of people with chronic migraine, all of whom had tried and discontinued multiple migraine preventatives including onabotulinumtoxinA, and who also had a high prevalence of depression and medication overuse. We feel our cohort is representative of the small minority of patients with difficult-to-treat chronic migraine who do not respond to readily available therapies (ie non-CGRP treatments). We found significant improvements in terms of both the number of headache days and their functional impact. Placebo-controlled trials in this population are required to confirm these findings.

## Supplementary Information

**Additional file 1: Supplementary Figure 1.** Q plots showing change in number of red days against the expected normal at 3 months, 6 months and 9 months compared to baseline.

## Data Availability

The datasets used and analysed during the current study are available from the corresponding author on reasonable request.

## Abbreviations

BoNTA: Onabotulinumtoxin A
HIT-6: Headache impact test-6
PHQ-9: Patient health questionnaire-9
PDI: Pain disability index
CGRP: Calcitonin gene-related peptide
FDA: Food and Drug Administration
EMA: European Medicines Agency
NICE: National Institute of Clinical Excellence
EHF: European Headache Federation
