# Supplementary figures and images for: Improvements in pain, medication use and quality of life in onabotulinumtoxinA-resistant chronic migraine patients following erenumab treatment – real world outcomes
Source: J Headache Pain. 2021 Jan 9;22(1):5. doi: 10.1186/s10194-020-01214-2 (PMC7797151; doi:10.1186/s10194-020-01214-2)

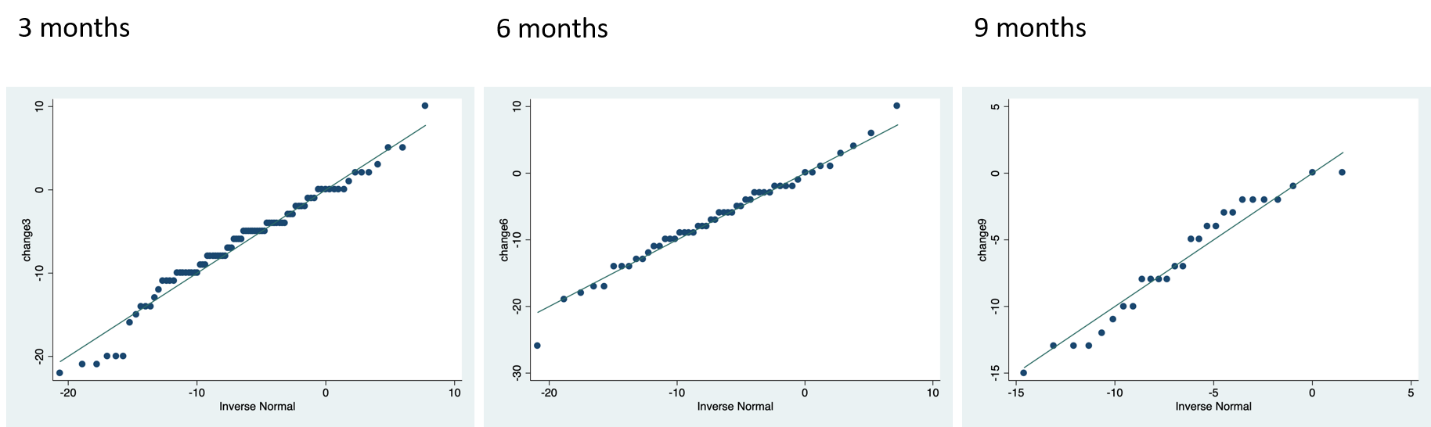

Supplement: Supplementary file 1 — Additional file 1: Supplementary Figure 1. Q plots showing change in number of red days against the expected normal at 3 months, 6 months and 9 months compared to baseline. [file 10194_2020_1214_MOESM1_ESM.tif]
